# Cryostructuring of Polymeric Systems. 50.† Cryogels and Cryotropic Gel-Formation: Terms and Definitions

**DOI:** 10.3390/gels4030077

**Published:** 2018-09-10

**Authors:** Vladimir I. Lozinsky

**Affiliations:** A. N. Nesmeyanov Institute of Organoelement Compounds, Russian Academy of Sciences, Vavilov Street 28, 119991 Moscow, Russia; loz@ineos.ac.ru

**Keywords:** cryogel(s), cryostructurate(s), cryotropic gel-formation, cryostructuring, macroporosity, terms, definitions

## Abstract

A variety of cryogenically-structured polymeric materials are of significant scientific and applied interest in various areas. However, in spite of considerable attention to these materials and intensive elaboration of their new examples, as well as the impressive growth in the number of the publications and patents on this topic over the past two decades, a marked variability of the used terminology and definitions is frequently met with in the papers, reviews, theses, patents, conference presentations, advertising materials and so forth. Therefore, the aim of this brief communication is to specify the basic terms and definitions in the particular field of macromolecular science.

A variety of cryogenically-structured polymeric materials are now known to be of significant scientific and applied interest in various areas, including both such delicate disciplines as medicine, biology, cell/tissue engineering, and biotechnology, and the more scaled fields like food technology, ecology, construction in permanent frost regions, etc. [1,2,3,4,5,6,7,8,9,10,11,12,13,14,15,16,17,18,19,20,21,22,23,24]. However, despite considerable attention (scientific and applied) to these materials, intensive elaboration of their new examples, and impressive growth of the publication and patent activities within this topic over the past two decades, a marked variability of the used terms and definitions is frequently met with in the papers, reviews, theses, patents, conference presentations, advertising materials and so forth.

Since the subjects of this Special Issue of *Gels* are so-called cryogels and cryostructurates, as well as the processes of their formation, i.e., the respective cryotropic gel-formation and cryostructuring processes, the aim of this brief communication is to specify the terms and definitions in the particular field of macromolecular science.

## 1. Cryogels and Cryostructurates

**Figure 1:**
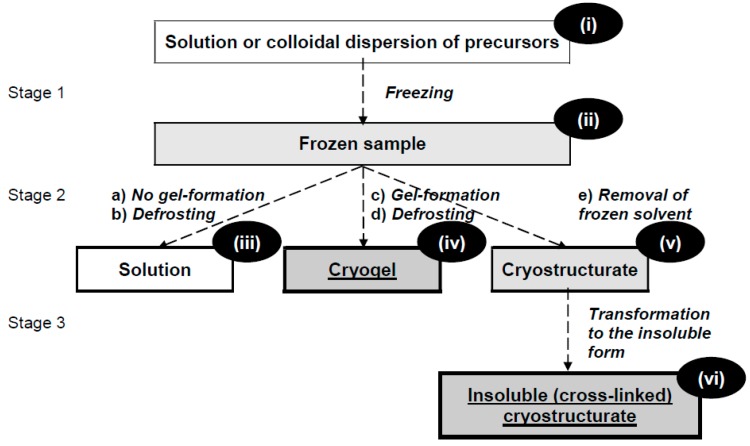
Principal scheme of the freezing-induced gel-formation and cryostructuring of solutions or colloidal dispersions of the respective precursors.

The meaning of complex words (e.g., cryogels or cryostructurates) that include the syllable ‘cryo’ (from the Greek κρύος (kryos) meaning frost) [4] is rather clear: These are the polymeric gels and structurates (texturates—the term used in food science) formed via cryogenic processing. In this context, the general idea, which visually reflects the definitions of cryogels and cryostructurates, is illustrated by the principal scheme in Figure 1.

If some molecular or colloid solution of the precursors (**i**) is being non-deeply frozen (Stage 1), and no gelation proceeds in such a frozen system (**ii**), then, after its thawing (Stage 2), a solution (**iii**) is produced again. It means that if, under the frozen conditions, no arising of the sufficiently stable junction knots of the 3D polymeric network occurs, no cryogenically-structured material can be obtained at the end of the system defrosting. In turn, if some gel-formation processes are able to proceed in a frozen sample (**ii**), its further incubation in the frost-bound state and subsequent thawing (Stage 2) yield to the macroporous gel matrix, which is precisely described by the term cryogel (**iv**) [4,8,15,25,26]. Therefore, simply speaking, cryogels are the macroporous polymeric gels that were formed in the moderately-frozen “solvent–precursors” systems [25]. 

The removal of the frozen solvent crystals from the frost-bound system (**ii**) at Stage 2e, for instance, freeze-drying will give rise to the “primary” spongy cryostructurate (**iv**). The latter one is soluble, but it can be transformed into insoluble matter using the appropriate chemical or radiation cross-linking (Stage 3) [4,25], thus resulting in the insoluble (cross-linked) “final” cryostructurate (**vi**). 

**Figure 2:**
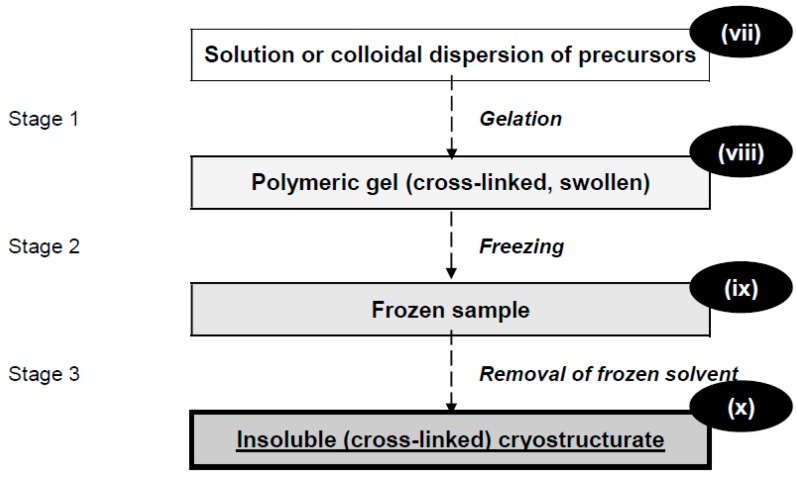
The diagram of the pre-formed gel matter cryostructuring.

Yet another type of polymeric cryostructurates is also known; the pathway for their preparation is demonstrated in Figure 2. 

In this case the initial solution (**vii**) contains the precursors in the amount sufficient for gel-formation at some positive temperatures, i.e., the concentration of precursors in the feed system is higher than the critical concentration of gelation. Upon the completion of such gelation (Stage 1) the resultant swollen gel (**viii**) is frozen (Stage 2). The crystallized phase is then removed from the frozen sample (**ix**) during Stage 3 either via freeze-drying or by the cryoextraction technique. This sequence of operations leads to the final macroporous cryostructurate (**x**). Its polymeric phase is being cured by the same covalent or non-covalent links as in the gel sample (**ix**) prior to its cryogenic treatment. A similar variant of this procedure is the swelling of the cross-linked polymeric network, which has been initially prepared in the absence of a solvent, and thereafter the swollen gel is processed like the swollen gel (**viii**). Freezing of the polymer solution and the swollen gel occurs at the same temperature, as a rule, non-identically, with different amounts, shapes and sizes of the solvent crystals being formed. It is so, since the polymer network of a gel interferes with the growth of the crystals [27,28,29]. Therefore, it is evident that the properties and the macroporous morphology of the cryostructurates (**v**) (Figure 1) and (**x**) (Figure 2) would differ despite the formal similarity of their compositions and the cross-linking extent, as well as the identical cryogenic processing conditions.

**Figure 3:**
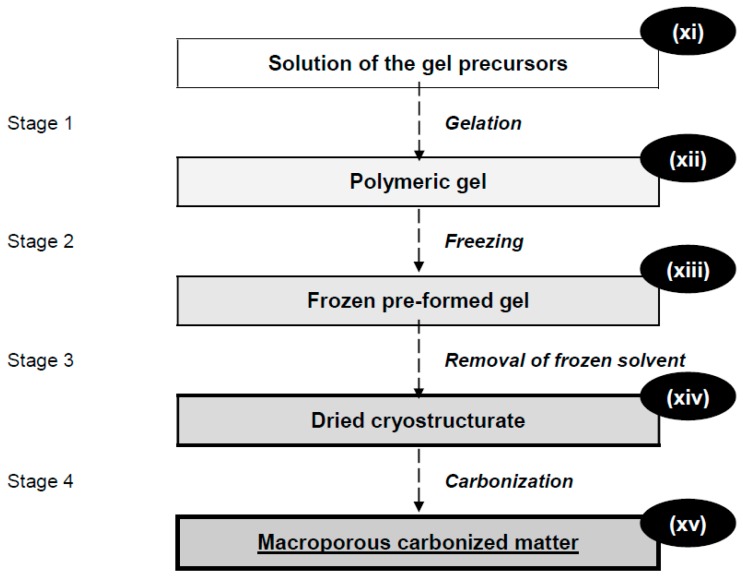
The scheme of the preparation of macroporous carbonized matrices.

The next case, which is of sufficient significance to be considered herein, is the preparation of the so-called ‘carbon cryogels’ [30,31]. These activated-carbon-like matrices are now rather popular for their use as electronic materials, absorbents, functional fillers in various composites. However, the application of the term ‘cryogels’ to them is of serious doubt. First of all, no cryotropic gel-formation is involved in their synthesis (Figure 3), where the usual cross-linked organogel (**xii**) is initially produced (Stage 1) without any freezing. Only then such “pre-formed” gel is frozen (Stage 2) and freeze-dried (Stage 3). Thereafter the resultant dry cryostructurate (**xiv**) is subjected to high-temperature carbonization (Stage 4), thus yielding the macroporous carbonized matter (**xv**). Secondly, such matter is not a gel at all, since it does not contain any solvate liquid, and, in fact, these materials consist only of the structured carbon (or, most probably, coal), which is incapable of swelling in either solvent, whereas the ability to swell is the characteristic feature of all gels in general. Therefore, it is thought that there are no scientifically-grounded reasons to call such cryogenically-structured carbon-based matrices by the term “cryogels”. 

Similar concerns about non-adequately used terminology also relate to the following now-known matters: (i) Cosmetic gels (commercial name “Cryogel^®^” [32]) that cause a cooling-down sensation after being applied to human skin; such gelatin-based jelly-like cream is fabricated without any cryogenic structuring. (ii) Flexible aerogel-based material marketed as “Cryogel^®^ Z”, which is used as a blanket insulation for the cryogenic apparatus [33]; the preparation of this material also does not involve any technique for cryogenic structuring. (iii) Protein gel-like coagulates formed at reduced positive temperatures, i.e., without any freeze-thaw influence, in the blood plasma taken from patients with immune diseases [34]. All the above materials do not relate either to the cryogels or to the cryostructurates. 

The term polymeric cryogel was proposed more than 30 years ago [35], and, according to the Web of Science data, more than 1900 papers have been published on this topic; the quotation amount for the respective publications exceeds 30,000 [36], and the integral quantity of the international plus national patents can hardly be estimated. This fact evidently demonstrates the scientific and applied importance of such gel materials and insists on the implementation of well-defined terminology for their description and discussion. 

## 2. Cryotropic Gel-Formation and Cryostructuring

For the designation of processes that result in the preparation of the polymeric cryogels and cryostructurates, the terms cryotropic gel-formation (cryogelation) and cryostructuring (cryostructuration), respectively, are used most frequently [4,25,26,37]. “Cryotropic” (from the Greek τροπικός (tropikos)—changed) means “the changes” caused (induced) by the cryogenic influence. Both types of the above processes, namely, cryotropic gel-formation and cryostructuration (Figure 1 and Figure 2), must include such key “events” as the ‘liquid–solid’ phase transition of the low-molecular solvent, i.e., its crystallization (but not the vitrification), upon a non-deep freezing of the feed system. In turn, the solvent crystallization induces significant increase in the solute’s concentration within the volume of the so-called unfrozen liquid microphase (UFLMP), i.e., in the regions remaining yet unfrozen in the bulk of the macroscopically frost-bound sample [38]. Such cryoconcentrating effect is the main driving force for the cryotropic gel-formation in spite of the reduced temperature and the very high viscosity of the UFLMP [4,25,26,37]. In the majority of known cases of the cryotropic gel-formation the UFLMP exists within a certain range of the “moderate” minus temperatures, not lower than 20–30 °C below the freezing point of a neat solvent. The latter one can be aqueous as in the case of water-compatible precursors or a crystallizable organic solvent, as in the case of the organosoluble precursors. The presence of such an unfrozen liquid microphase provides a mobile medium for the molecular/segmental movements and intermolecular interactions that are required for the generation of the 3D-network-knots in forming cryogels.

On the other hand, in the case of cryostructurates preparation, when no cryotropic gel-formation occurs, the requirements of freezing temperatures are not limited by the range of UFLMP existence. Here, the main condition is the necessity to freeze the system to be textured cryogenically, and the properties of the final cryostructurates ((**vi**), Figure 1 or (**x**), Figure 2) are stipulated in general by the initial concentration of the precursors and the freezing regime [39,40].

Similar to the conventional gels formed at positive temperatures, cryogels can be prepared starting from both the low-molecular (monomeric) and the polymeric precursors [4,25,26,37]. In the former case—via the cross-linking polymerization or polycondensation in the moderately-frozen media; in the latter case—via either covalent, or physical, or ionic cross-linking of the respective macromolecules in the non-deeply-frozen gelling system (Stage 2c, Figure 1). 

Regarding the nature of interchain links in the network’s junction knots, the cryogels, analogously to the conventional gels, can be classified as the covalent (cross-linked chemically), the non-covalent (physical) and the ionically cross-linked matrices [4,26]. With that, the cross-linked cryostructurates (**vi**) (Figure 1) and (**x**) (Figure 2) are known to be mainly chemically and ionically cross-linked polymeric matrices (e.g., see References [39,40,41,42]). 

In the common sense, such a term as ‘cryostructuring’ is a more general notion than the ‘cryotropic gel-formation’, since the freezing-caused structuration occurs in both these cases. However, in order to emphasize the specificity of the processes that result in the cryogels directly, the usage of the latter term, i.e., ‘cryotropic gel-formation’, is thought to be preferable. 

Two basic types of cryogels and cryostructurates can be obtained depending on the properties of the initial system and the cryogenic processing conditions: (i) the macroporous matrices with the pore cross-section over the range from ~0.1 to ~10 µm, and (ii) the supermacroporous or wide-porous spongy matrices with the pores of tens and even hundreds of micrometers [15,20]. It is clear that such classification is rather conventional, since the cryogenically-structured polymeric materials with the intermediate-size pores or with the hierarchical porosity are frequently met as well. Nonetheless, such simple classification is convenient enough, at least, for the qualitative description of the macroporous morphology of various cryogels and cryostructurates. The macroporosity is their characteristic feature. In general, the size and the shape of the respective large pores depend on many factors. The main factors are as follows: The precursors’ nature and concentration, the solvent used and its cryoscopic properties, the presence and amount of foreign solutes or disperse fillers, as well as the thermal regimes of cryogenic processing, namely, the cooling rate during freezing, the freezing temperature itself, frozen storage duration, the rate of the frozen samples heating for their thawing, the number of the freeze-thaw cycles (the latter two parameters are of especial significance for the physical cryogels like the poly(vinyl alcohol)-based ones [26,43,44,45,46,47,48,49]). 

Yet another specific peculiarity of the macroporous morphology of the polymeric cryogels and cryostructurates is the interconnected character of their porosity [1,4,25,26,37,43,49,50,51], providing that no special methods for the directed freezing are applied. The interconnections of macropores arise owing to the tight contacts of facets of the growing solvent polycrystals together upon the initial system freezing. Further, in the course of the frozen sample thawing, these contacts are transformed into ‘interlinks’ between the macropores. 

Typical examples of macroporous cryogenically-structured polymeric matrices are the aforementioned non-covalent poly(vinyl alcohol) cryogels [26,43,44,45,46,47,48,49,52,53,54], the amylopectin-based cryogels [55], certain locust-bean-gum-based cryogels [56]. Some well-known examples of the wide-porous/supermacroporous cryogels and cryostructurates are those based on the cross-linked poly(acrylamide) [57,58,59,60,61,62,63], thermoresponsive cross-linked poly(*N*,*N*-diethylacrylamide) [64], poly(N-isopropylacrylamide) [65,66,67,68,69,70] and poly(*N*-vinylcaprolactram) [71], radiation- and chemically-polymerized 2-hydroxyethyl methacrylate [72,73,74,75], covalently cross-linked proteins [39,40,76,77,78,79,80], polysaccharides [21,41,55,56,81,82,83,84,85,86,87] and nucleic acids [88,89], various synthetic high polymers [4,21,35,37,90,91,92,93,94], as well as numerous physical and ionic cryogels and cryostructurates [4,12,56,95,96,97,98,99,100,101,102,103], etc. All these polymeric materials have a system of interconnected large pores. This property makes such materials very promising in view of the application possibilities in various fields, especially materials of biomedical and biotechnological interest [4,5,7,8,9,10,11,13,15,16,19,20,21,22,23,24,40,41,103,104,105,106,107,108,109,110,111,112,113,114,115,116,117,118,119,120,121,122,123,124,125,126,127,128,129,130,131,132]. 

In some cases, the term ‘superporous’ is also used in relation to cryogenically-structured polymeric matrices. It is thought that such definition without indication of the size of the pores (as in the term ‘supermacroporous’) is not sufficiently exact (the particular references are not given here because of ethical reasons). The prefix ‘super’ in respect of the porosity of the cryogels or cryostructurates may imply that these materials are highly porous (extra-porous), i.e., they contain a very large number of pores, but it can be so only if such pores are very small. 

The last terminological remark in this communication concerns the applicability of the terms ‘monolith’ and ‘monolithic’ in regard to the wide-porous sponge-like soft cryogels and cryostructurates. These terms have become very popular during the past two decades in connection with the developments of so-called ‘monolithic stationary phases’ for various types of liquid chromatography (certain beds inside the respective columns are able to function at elevated pressure) [133,134,135]. Later the terms ‘monolith’ and ‘monolithic’ were simply “transferred” by some authors (the particular references are not quoted here as well) for the particular cryogel-type soft matrices used as the chromatographic-like beds. The objections for such terminological transfer have been already expressed elsewhere [8]; therefore, they are simply cited once more:

Unfortunately, terminological imprecision which distorts the essence of the considered problems are often met in the literature concerning the wide-pore cryogels. For instance, the term ‘monoliths’ is acquired popularity in the recent time, although the monolith (from the Greek word μονολίτης (monolithis)—a single stone, whole stone lump) can rather be referred to a block of the well-known homophase poly(acrylamide) gel, which is widely used as a medium for the electrophoretic separation of substances, rather than to the supermacroporous heterophase cryogel. The use of the term ‘monolith’ with respect to a soft spongy material, the liquid from which can simply be pressed out under a small mechanical load, seems quite unsubstantiated. If the pressed-out sample, which has lost its primary shape due to this, is again placed in the original solvent, then the cryogel very rapidly swells absorbing the liquid through the system of interconnected capillaries, and recovers the shape completely. Therefore, its elastic properties in the swollen state are mainly determined not by the rigidity of the polymeric framework, as some authors surprisingly believe, but by the swelling pressure and capillary forces, which have been shown for the non-covalent poly(galactomannan) and chemically cross-linked poly(isobutylene) cryogels.

The last two examples were described in References [56,91,92,93]. In them, the term ‘monoliths’ was not used and for the poly(isobutylene) cryogels it was shown that these matrices “can be compressed up to about 100% strain without any crack development, during which the total liquid inside the gel is squeezed out.” It is clear that such properties are not absolutely characteristic, of course, of the real monoliths even if they contain some pores. 

## References

[1] Lawrence R., Consolacion F., Jelen P. (1986). Formation of structured protein foods by freeze texturization. Food Technol..

[2] Lozinsky V.I., Vakula A.V., Zubov A.L. (1992). Application of poly(vinyl alcohol) cryogels in biotechnology. IV. Literature data overview. Sov. Biotechnol..

[3] Chu K.C., Rutt B.K. (1997). Poly(vinyl alcohol) cryogel: An ideal phantom material for MR studies of arterial flow and elasticity. Magn. Reson. Med..

[4] Lozinsky V.I. (2002). Cryogels on the basis of natural and synthetic polymers: Preparation, properties and areas of implementation. Russ. Chem. Rev..

[5] Lozinsky V.I., Galaev I.Y., Plieva F.M., Savina I.N., Jungvid H., Mattiasson B. (2003). Polymeric cryogels as promising materials of biotechnological interest. Trends Biotechnol..

[6] Altunina L.K., Kuvshinov V.A., Dolgikh S.N., Lombardi S., Altunina L.K., Beaubien S.E. (2006). Cryogels—A promising material for underground works in permafrost. Advances in Geological Storage of Carbon Dioxide.

[7] Daniak M.B., Galaev I.Y., Kumar A., Plieva F.M., Mattiasson B. (2007). Chromatography of living cells using supermacroporous hydrogels, cryogels. Adv. Biochem. Eng. Biotechnol..

[8] Lozinsky V.I. (2008). New generation of macroporous and supermacroporous materials of biotechnological interest—Polymeric cryogels. Russ. Chem. Bull..

[9] Hoskins P.R. (2008). Simulation and validation of arterial ultrasound imagining and blood flow. Ultrasound Med. Biol..

[10] Plieva F.M., Galaev I.Y., Noppe W., Mattiasson B. (2008). Cryogel applications in microbiology. Trends Microbiol..

[11] Shoichet M.S. (2010). Polymer scaffolds for biomaterials applications. Macromolecules.

[12] Qian L., Zhang H. (2011). Controlled freezing and freeze drying: A versatile route for porous and micro-/nano-structured materials. J. Chem. Technol. Biotechnol..

[13] Baker M.I., Walsh S.P., Schwatz Z., Boyan B.D. (2012). A review of polyvinyl alcohol and its uses in cartilage and orthopedic applications. J. Biomed. Mater. Res. B.

[14] Geidobler R., Winter G. (2013). Controlled ice nucleation in the field of freeze-drying: Fundamentals and technology review. Eur. J. Pharm. Biopharm..

[15] Okay O. (2014). Polymeric Cryogels: Macroporous Gels with Remarkable Properties.

[16] Liu C., Tong G., Chen C., Tan Z., Quan C., Zhang C. (2014). Polymeric cryogel: Preparation, properties and biomedical applications. Progr. Chem..

[17] Altunina L.K., Fufaeva M.S., Filatov D.A., Scarovskaya L.I., Rozhdestvenskii E.A., Gan-Erdene T. (2014). Effect of cryogel on soil properties. Euroasian Soil Sci..

[18] Vasiliev N.K., Pronk A.D.C., Shatalina I.N., Janssen F.H.M.E., Houben R.W.G. (2015). A review on the development of reinforced ice for use as a building material in cold regions. Cold Reg. Sci. Technol..

[19] Choudhury S., Connolly D., White B. (2015). Supermacroporous polyHIPE and cryogel monolithic materials as stationary phases in separation science: A review. Anal. Methods.

[20] Kumar A. (2016). Supermacroporous Cryogels: Biomedical and Biotechnological Applications.

[21] Jiang S., Agarwal S., Greiner A. (2017). Low-density open cellular sponges as functional materials. Angew. Chem. Int. Ed..

[22] Hixon K.R., Lu T., Sell S.A. (2017). A comprehensive review of cryogels and their roles in tissue engineering applications. Acta Biomater..

[23] Privar Y., Malakhova I., Pestov A., Fedorets A., Azarova Y., Schwarz S., Bratskaya S. (2018). Polyethyleneimine cryogels for metal ions sorption. Chem. Eng. J..

[24] Nadgorny M., Collins J., Xiao Z., Scales P.J., Connal L.A. (2018). 3D-printing of dynamic self-healing cryogels with tunable properties. Polym. Chem..

[25] Lozinsky V.I. (2014). A breif history of polymeric cryogels. Adv. Polym. Sci..

[26] Lozinsky V.I., Okay O. (2014). Basic principles of cryotropic gelation. Adv. Polym. Sci..

[27] Kuhn B., Peterli E., Majer H. (1955). Freezing point depression of gels produced by high polymer networks. J. Polym. Sci..

[28] Oikawa H., Murakami K. (1989). Relationship between swollen network structure of rabber vulcanizates and mechanism of freezing point depression of swelling solvent. J. Macromol. Sci. Phys..

[29] Henisch H.K. (1996). Crystal Growth in Gels.

[30] Tamon H., Ishizaka H., Yamamoto T., Suzuki T. (1999). Preparation of mesoporous carbon by freeze drying. Carbon.

[31] Job N., Thery A., Pirard R., Marien J., Kocon L., Rouzaud J.N., Beguin F., Pirard J.P. (2005). Carbon aerogels, cryogels and xerogels: Influence of the drying method on the textural properties of porous carbon materials. Carbon.

[32] Cryogel®. http://www.gelatin.com/en/cold-solubles/product-range/cryogel.

[33] Cryogel® Z. https://www.aerogel.com/products-and-solutions/cryogel-z.

[34] Miyamoto K., Tokita K., Miashita K., Sakashita E. (2001). Cryogelation in vitro. Int. J. Biol. Macromol..

[35] Lozinsky V.I., Vainerman E.S., Korotaeva G.F., Rogozhin S.V. (1984). Study of cryostructurization of polymer systems. III. Cryostructurization in organic media. Coll. Polym. Sci..

[36] Web of Science. http://apps.webofknowledge.com/CitationReport.do?product=WOS&search_mode=CitationReport&SID=F2nYMNzOJrWECQJdDi5&page=1&cr_pqid=4&viewType=summary&colName=WOS.

[37] Okay O., Lozinsky V.I. (2014). Synthesis, structure-property relationships of cryogels. Adv. Polym. Sci..

[38] Sergeev G.B., Batyuk V.A. (1976). Reactions in frozen multicomponent systems. Russ. Chem. Revs..

[39] Rodionov I.A., Grinberg N.V., Burova T.V., Grinberg V.Y., Shabatina T.I., Lozinsky V.I. (2017). Cryostructuring of polymer systems. 44. Freeze-dried and then chemically cross-linked wide porous cryostructurates based on serum albumin. e-Polymers.

[40] Lozinsky V.I., Kulakova V.K., Ivanov R.V., Petrenko A.Y., Rogulska O.Y., Petrenko Y.A. (2018). Cryostructuring of polymer systems. 47. Preparation of wide porous gelatin-based cryostructurates in sterilizing organic media and assessment of the suitability of thus formed matrices as spongy scaffolds for 3D cell culturing. e-Polymers.

[41] Petrenko Y.A., Ivanov R.V., Petrenko A.Y., Lozinsky V.I. (2011). Coupling of gelatin to inner surfaces of pore walls in spongy alginate-based scaffolds facilitates the adhesion, growth and differentiation of human bone marrow mesenchymal stromal cells. J. Mater. Sci. Mater. Med..

[42] Brovko O.S., Palamarchuk I.A., Val’chuk N.A., Chukhchin D.G., Bogolitsyn K.G., Boitsova T.A. (2017). Gels of sodium alginate—Chitosan interpolyelectrolyte complexes. Russ. J. Phys. Chem. Ser. A.

[43] Lozinsky V.I. (1998). Cryotropic gelation of poly(vinyl alcohol) solutions. Russ. Chem. Rev..

[44] Willcox P.J., Howie D.W., Schmidt-Rohr K., Hoagland D.A., Gido S.P., Pudjijanto S., Kleiner W., Venkatraman S. (1999). Microstructure of poly(vinyl alcohol) hydrogels produced by freeze/thaw cycling. J. Polym. Sci. B Polym. Phys..

[45] Hassan C.M., Peppas N.A. (2000). Structure and applications of poly(vinyl alcohol) hydrogels produced by conventional crosslinking or by freezing/thawing methods. Adv. Polym. Sci..

[46] Hassan C.M., Peppas N.A. (2000). Structure and morphology of freeze/thawed PVA hydrogels. Macromolecules.

[47] Hatakeyama T., Uno J., Yamada C., Kishi A., Hatakeyama H. (2005). Gel-sol transition of poly(vinyl alcohol) hydrogels formed by freezing and thawing. Thermochim. Acta.

[48] Lozinsky V.I., Damshkaln L.G., Shaskol’skii B.L., Babushkina T.A., Kurochkin I.N., Kurochkin I.I. (2007). Study of cryostructuring of polymer systems. 27. Physicochemical properties of poly(vinyl alcohol) cryogels and features of their macroporous morphology. Colloid J..

[49] Lozinsky V.I., Damshkaln L.G., Kurochkin I.N., Kurochkin I.I. (2008). Study of cryostructuring of polymer systems. 28. Physicochemical and morphological properties of poly(vinyl alcohol) cryogels formed via multiple freezing-thawing technique. Colloid J..

[50] Okay O. (2000). Macroporous copolymer networks. Progr. Polym. Sci..

[51] Tanthapanichakoon W., Tamon H., Nakagawa K., Charinpanitkul T. (2013). Synthesis of porous materials and their microstructural control through ice templating. Eng. J..

[52] Holloway J.L., Lowman A.M., Palmese G.R. (2013). The role of crystallization and phase separation in the formation of physically cross-lonked PVA hydrogels. Soft Matter.

[53] De Rosa C., Auriemma F., Girolamo R.D. (2014). Kinetic analysis of cryotropic gelation of poly(vinyl alcohol)/water solutions by small-angle neutron scattering. Adv. Polym. Sci..

[54] Suzuki A., Sasaki S. (2015). Swelling and mechanical properties of physically crosslinked poly(vinyl alcohol) hydrogels. J. Eng. Med..

[55] Lozinsky V.I., Damshkaln L.G., Brown C.R.T., Norton I.T. (2000). Study of cryostructuration of polymer systems. XVIII. Freeze-thaw-influence on water-solubilized artificial mixtures of amylopectin and amylose. J. Appl. Polym. Sci..

[56] Lozinsky V.I., Damshkaln L.G., Brown C.R.T., Norton I.T. (2000). Study of cryostructuring of polymer systems. XIX. On the nature of intermolecular links in the cryogels of locust bean gum. Polym. Int..

[57] Lozinsky V.I., Vainerman E.S., Titova E.F., Belavtseva E.M., Rogozhin S.V. (1984). Study of cryostructurization of polymer systems. IV. Cryostructurization of the system: Solvent—Vinyl monomer—Divinyl monomer—Initiator of polymerization. Coll. Polym. Sci..

[58] Belavtseva E.M., Titova E.F., Lozinsky V.I., Vainerman E.S., Rogozhin S.V. (1984). Study of cryostructurization of polymer systems. V. Electron microscopic studies of cross-linked polyacrylamide cryogels. Coll. Polym. Sci..

[59] Gusev D.G., Lozinsky V.I., Bakhmutov V.I. (1993). Study of cryostructurization of polymer systems. X. ^1^H- and ^2^H-NMR studies of the formation of crosslinked polyacrylamide cryogels. Eur. Polym. J..

[60] Plieva F.M., Karlsson M., Aguilar M.-R., Gomez D., Mikhalovsky S., Galaev I.Y. (2005). Pore structure in supermacroporous polyacrylamide based cryogels. Soft Matter.

[61] Ivanov R.V., Lozinsky V.I., Noh S.K., Han S.S., Lyoo W.S. (2007). Preparation and characterization of polyacrylamide cryogels produced from a high-molecular weight precursor. I. Influence of the reaction temperature and concentration of the cross-linking agent. J. Appl. Polym. Sci..

[62] Ivanov R.V., Lozinsky V.I., Noh S.K., Lee Y.R., Han S.S., Lyoo W.S. (2008). Preparation and characterization of polyacrylamide cryogels produced from a high-molecular-weight precursor. II. The influence of the molecular weight of the polymeric precursor. J. Appl. Polym. Sci..

[63] Carvalho B.M.A., Da Silva S.L., Da Silva L.H.M., Minim V.P.R., Da Silva M.C.H., Carvalho L.M., Minim L.A. (2014). Cryogel poly(acrylamide): Synthesis, structure and applications. Sep. Purif. Rev..

[64] Lozinsky V.I., Kalinina E.V., Grinberg V.Y., Grinberg N.V., Chupov V.A., Plate N.A. (1997). Thermoresponsive cryogels based on cross-linked poly(*N*,*N*-diethylacrylamide). Polym. Sci. Ser. A.

[65] Zhang X.-Z., Zhuo R.-X. (1999). A novel method to prepare a fast responsive, temperature-sensitive poly(*N*-isopropylacrylamide) hydrogel. Macromol. Rapid Commun..

[66] Zhang X.-Z., Zhuo R.-X. (1999). Preparation of fast responsive, temperature-sensitive poly(*N*-isopropylacrylamide) hydrogel. Macromol. Chem. Phys..

[67] Srivastava A., Jain E., Kumar A. (2007). The physical characterization of supermacroporous poly(*N*-isopropylacrylamide) cryogel: Mechanical strength and swelling/de-swellining kinetics. Mater. Sci. Eng. A.

[68] Strachotova B., Strachota A., Uchman M., Šlouf M., Brus J., Pleštil L., Matĕjka L. (2007). Super porous organic-inorganic poly(*N*-isopropylacrylamide)-based hydrogel with a very fast temperature response. Polymer.

[69] Chalal M., Ehrburger-Dolle F., Morfin I., Vial J.C., de Armas M.R.A., Roman J.S., Bülgen N., Pişkin E., Ziane O., Caslegno R. (2009). Imaging the structure of macroporous hydrogels by two-proton fluorescence microscopy. Macromolecules.

[70] Sahiner N. (2018). Super macroporous poly(*N*-isopropyl acrylamide) cryogel for separation purposes. Polym. Adv. Technol..

[71] Srivastava A., Kumar A. (2010). Thermoresposive poly(*N*-vinylcaprolactam) cryogels: Synthesis and its biophysical evaluation for tissue engineering applications. J. Mater. Sci. Mater. Med..

[72] Kaetsu I. (1993). Radiation synthesis of polymeric materials for biomedical and biochemical applications. Adv. Polym. Sci..

[73] Kumakura M. (2001). Preparation method of porous polymer materials by radiation technique and its application. Polym. Adv. Technol..

[74] Pavlova L.A., Kastelyanos-Dominges O.M. (1996). Preparation of cryogels by polymerization of 2-hydroxyethyl methacrylate in the presence of sodium persulfate-*N*,*N*,*N*′,*N*′-tetramethylethylenediamine initiating system. Russ. J. Appl. Chem..

[75] Perçin I., Saglar E., Yavuz H., Aksoz E., Denizli A. (2011). Poly(hydroxyethyl methacrylate) based affinity **cryogel** for plasmid DNA purification. Int. J. Biol. Macromol..

[76] Elowsson L., Kirsebom H., Carmignac V., Durbeej M., Mattiasson B. (2012). Porous protein-based scaffolds prepared through freezing as potential scaffolds for tissue engineering. J. Mater. Sci. Mater. Med..

[77] Rodionov I.A., Grinberg N.V., Burova T.V., Grinberg V.Y., Lozinsky V.I. (2015). Cryostructuring of polymeric systems. 40. Proteinaceous wide-pore cryogels generated by the action of denaturant/reductant mixtures on bovine serum albumin in moderately-frozen aqueous media. Soft Matter.

[78] Van Vlierberghe S. (2016). Crosslinking strategies for porous gelatin scaffolds. J. Mater. Sci..

[79] Ni N., Duquette D., Dumont M.-J. (2017). Synthesis and characterization of zein-based cryogels and their potential as diesel fuel absorbent. Eur. Polym. J..

[80] Wu Q., Lindh V.H., Johansson E., Olsson R.T., Hedenqvist M.S. (2017). Freeze-dried wheat gluten biofoams: Scaling up with water welding. Ind. Crops Prod..

[81] Lozinsky V.I., Vainerman E.S., Rogozhin S.V. (1982). Study of cryostructurization of polymer systems. II. The influence of freezing of reacting mass on the properties of products in the preparation of covalently cross-linked gels. Coll. Polym. Sci..

[82] Kirsebom H., Aguilar M.R., San Roman J., Fernandez M., Prieto M.A., Bondar B. (2007). Macroporous scaffolds based on chitosan and bioactive molecules. J. Bioact. Compat. Polym..

[83] Lozinsky V.I., Damshkaln L.G., Bloch K.O., Vardi P., Grinberg N.V., Burova T.V., Grinberg V.Y. (2008). Cryostructuring of polymer systems. XXIX. Preparation and characterization of supermacroporous (spongy) agarose-based cryogels used as three-dimensional scaffolds for culturing insulin-producing cell aggregates. J. Appl. Polym. Sci..

[84] Plieva F.M., Galaev I.Y., Mattiasson B., Mattiasson B., Kumar A., Galaev I.Y. (2010). Macroporous polysaccharide gels. Macroporous Polymers: Production, Properties and Biotechnological/Biomedical Applications.

[85] Reichelt S., Becher J., Weisser J., Prager A., Decker U., Möller S., Berg A., Schnabelrauch M. (2014). Biocompatible polysaccharide-based cryogels. Mater. Sci. Eng. C.

[86] Zhao Y., Shen W., Chen Z., Wu T. (2016). Freeze-thaw-induced gelation of alginates. Carbohydr. Polym..

[87] Georgiev G.L., Trzebicka B., Kostova B., Petrov P.D. (2017). Super-macroporous dextran cryogels via UV-induced crosslinking: Synthesis and characterization. Polym. Int..

[88] Okay O. (2011). DNA Hydrogels: New Functional Soft Materials. J. Polym. Sci. Part B Polym. Phys..

[89] Karacan P., Okay O. (2013). Ethidium bromide binding to DNA cryogels. React. Funct. Polym..

[90] Lozinsky V.I., Golovina T.O., Gusev D.G. (2000). Study of cryostructuration of polymer systems. XIII. Some characteristic features of the behaviour of macromolecular thiols in frozen aqueous solutions. Polymer.

[91] Ceylan D., Okay O. (2007). Macroporous polyisobutylene gels: A novel tough organogel with superfast responsibility. Macromolecules.

[92] Oztoprak Z., Hekimoglu T., Karaturuk I., Tunkaboylu D.C., Okay O. (2014). Porous rubber cryogels: Effect of the gel preparation temperature. Polym. Bull..

[93] Karakutuk I., Okay O. (2010). Macroporous rabber gels as reusable sorbents for the removal of oil from surface waters. React. Funct. Polym..

[94] Petrov P., Utrata-Wesołek A., Trzebicka B., Tsvetanov C.B., Dworak A., Anioł J., Sieroń A. (2011). Biocompatible cryogels of thermosensitive polyglycidol derivatives with ultra-rapid swelling properties. Eur. Polym. J..

[95] Konstantinova N.R., Lozinsky V.I. (1997). Cryotropic gelation of ovalbumin solutions. Food Hydrocoll..

[96] Giannouli P., Morris E.R. (2003). Cryogelation of xantan. Food Hydrocoll..

[97] Zeira A., Nussinovitch A. (2004). Mechhanical properties of weak locust bean gum (LBG) gels under controlled rapid freeze-thawing. J. Texture Stud..

[98] Zhang H., Zhang F., Wu J. (2013). Physically crosslinked hydrogels from polysaccharides prepared by freeze-thaw technique. React. Func. Polym..

[99] Muthukumar T., Aravinthan A., Sharmila J., Kim N.S., Kim J.-H. (2016). Collagen/chitosan porous bone tissue engineering composite scaffold incorporated with *Ginseng* compound K. Carbohydr. Polym..

[100] De la Portilla F., Pereira S., Molero M., De Marco F., Perez-Puyana V., Guerrero A., Romero A. (2016). Microstructural, mechanical, and histological evaluation of modified alginate-based scaffolds. J. Biomed. Mater. Res. A.

[101] Vernaya O.V., Shabatin V.P., Nuzhdina A.V., Zvukova N.D., Khvatov D.I., Semenov A.M., Lozinsky V.I., Shabatina T.I., Mel’nikov M.Y. (2017). Cryochemical synthesis and antibacterial activity of hybrid nanocomposites of dioxydine with Ag and Cu nanoparticles entrapped in biopolymeric cryostructurates. Russ. Chem. Bull..

[102] Cassanelli M., Norton I., Mills T. (2018). Role of gellan gum microstructure in freeze drying and rehydration mechanisms. Food Hydrocoll..

[103] Sazhnev N.A., Drozdova M.G., Rodionov I.A., Kil’deeva N.R., Balabanova T.V., Markvicheva E.A., Lozinsky V.I. (2018). Preparation of chitosan cryostructurates with controlled porous morphology and their use as 3D-scaffolds for the cultivation of animal cells. Appl. Biochem. Microbiol..

[104] Varfolomeev S.D., Rainina E.I., Lozinsky V.I. (1992). Cryoimmobilized enzymes and cells in organic synthesis. Pure Appl. Chem..

[105] Lazzeri L. (1996). Progress in bioartificial polymeric materials. Trends Polym. Sci..

[106] Lozinsky V.I., Plieva F.M. (1998). Poly(vinyl alcohol) cryogels employed as matrices for cell immobilization. 3. Overview of recent research and developments. Enzyme Microb. Technol..

[107] Lozinsky V.I., Plieva F.M., Galaev I.Y., Mattiasson B. (2001). The potential of polymeric cryogels in bioseparation. Bioseparation.

[108] Surry K.J.M., Austin H.J.B., Fenster A., Peters T.M. (2004). Poly(vinyl alcohol) cryogel phantoms for use in ultrasound and MR imaging. Phys. Med. Biol..

[109] Plieva F.M., Galaev I.Y., Mattiasson B. (2007). Macroporous gels prepared at subzero temperatures as novel materials foe chromatography of particulate-containing fluids and cell culture applications. J. Sep. Sci..

[110] Kumar A., Bhardwaj A. (2008). Methods in cell separation for biomedical application: Cryogels as a new tool. Biomed. Mater..

[111] Gutiérrez M.C., Ferrer M.L., del Monte F. (2008). Ice-templated materials: Sophisticated structures exhibiting nhanced functionalities obtained after unidirectional freezing and ice-segregation-induced self-assembly. Chem. Mater..

[112] Alves M.H., Jensen B.E.B., Smith A.A.A., Zelikin A.N. (2011). Poly(vinyl alcohol) physical hydrogels: New vista on a long serving biomaterial. Macromol. Biosci..

[113] Gun’ko V.M., Savina I.N., Mikhalovsky S.V. (2013). Cryogels: Morphological, structural and adsorption characterization. Adv. Coll. Interface Sci..

[114] Henderson T.M.A., Ladewig K., Haylock D.N., McLean K.M., J’Connor A.J. (2013). Cryogels for biomedical applications. J. Mater. Chem. B.

[115] Mattiasson B. (2014). Cryogels for biotechnological applications. Adv. Polym. Sci..

[116] Wan W., Bannerman A.D., Yang L., Mak H. (2014). Poly(vinyl alcohol) cryogels for biomedical application. Adv. Polym. Sci..

[117] Dragan E.S. (2014). Design and applications of interpenetrating polymer network hydrogels. Chem. Eng. J..

[118] Cheng Q., Huang C., Tomsia A.P. (2017). Freeze casting for assembling bioinspired structural materials. Adv. Mater..

[119] Reichelt S. (2015). Introduction to macroporous cryogels. Methods Mol. Biol..

[120] Qi C., Yan X., Huang C., Melerzanov A., Du Y. (2015). Biomaterials as carrier and reactor for cell-based regenerative medicine. Protein Cell.

[121] Sedlačik T., Okar O.K., Studenovská H., Kotelnikov I., Kučka J., Konečná Z., Zikmund T., Kaiser J., Kose G.T., Rypáček F. (2018). Chondrogenik potential of macroporous biodegradable cryogels based on synthetic poly(α-amino acids). Soft Matter.

[122] Efremenko E.N., Lyagin I.V., Lozinsky V.I., Kumar A. (2016). Enzymatic biocatalysts immobilized on/in the cryogel-type carriers. Supermacroporous Cryogels: Biomedical and Biotechnological Applications.

[123] Andaç M., Galaev I.Y., Denizli A. (2016). Affinity based and molecularly imprinted cryogels: Applications in biomacromolecule purification. J. Chromatogr. B Anal. Technol. Biomed. Life Sci..

[124] Salgado C.L., Grenho L., Fernandez M.H., Colaço B.J., Monteiro F.J. (2016). Biodegradation, biocompatibility, and osteoconduction evaluation of collagen-nanohydroxyapatite cryogels for bone tissue regeneration. J. Biomed. Mater. Res. Part A.

[125] Gupta A., Bhat S., Chaudhari B.P., Gupta K.C., Tägil M., Zheng M.H., Kumar A., Lidgren L. (2017). Cell factory-derived bioactive molecules with polymeric cryogel scaffold enhance the repair of subchoronal cartilage defect in rabbits. J. Tissue Eng. Regen. Med..

[126] Shakya A.R., Kandalam U. (2017). Three-dimensional macroporous materials for tissue engineering of craniofacial bone. Br. J. Oral Maxillofac. Surg..

[127] Timofejeva A., D’Este M., Loca D. (2017). Calcium phosphate/polyvinyl alcohol composite hydrogels: A review on the freeze-thaw synthesis approach and applications in regenerative medicine. Eur. Polym. J..

[128] Çorman M.E. (2018). Poly-l-lysin mofified cryogels for efficient bilirubin removal from human plasma. Colloids Surfaces B Biointerfaces.

[129] Georgopoulou A., Papadogiannis F., Batsali A., Marakis J., Alpantaki K., Eliopolous A.G., Pontikoglou C., Chatzinikolaidou M. (2018). Chitosan/gelatin scaffolds support bone regeneration. J. Mater. Sci. Mater. Med..

[130] Kim I., Lee S.S., Bae S., Lee H., Hwang N.S. (2018). Heparin functionalized injectable cryogel with rapid shape recovery property for neovascularization. Biomacromolecules.

[131] Sheikh P.A., Singh A., Kumar A. (2018). Oxygen-releasing antioxidant cryogel scaffolds with sustained oxygen delivery for tissue engineering applications. ACS Appl. Mater. Interfaces.

[132] Zarrintaj P., Manouchehri S., Ahmadi Z., Seab M.R., Urbanska A.M., Kaplan D.L., Mozafari M. (2018). Agarose-based biomaterials for tissue engineering. Carbohydr. Polym..

[133] Svec F. (2010). Porous polymer monoliths: Amazingly wide variety of techniques enabling their preparation. J. Chromatogr. A.

[134] Acquah C., Moy C.K.S., Danquah M.K., Ongkudon C.M. (2016). Developments and characteristics of polymer monoliths for advanced LC bioscreening applications: A review. J. Chromatogr. B.

[135] Li Z., Rodriguez E., Azaria S., Pekarek A., Hage D.S. (2017). Affinity monolith chromatography: A review of general principles and applications. Electrophoresis.

